# Relationship Between Nutritional Indexes and Clinical Outcomes in Stroke Patients Undergoing Mechanical Thrombectomy

**DOI:** 10.3390/brainsci15070704

**Published:** 2025-06-30

**Authors:** Özgür Zülfükar Ertuğrul, Fırat Karaaslan, Reşit Yılmaz, Mehmet Cudi Tuncer

**Affiliations:** 1 Gazi Yaşargil Training and Research Hospital, Department of Radiology, University of Health Sciences, Diyarbakır 21090, Turkey; stenoz@hotmail.com; 2 Gazi Yaşargil Training and Research Hospital, Department of Neurology, University of Health Sciences, Diyarbakır 21090, Turkey; dr.frt1321@gmail.com (F.K.); dr.resityilmaz@gmail.com (R.Y.); 3 Faculty of Medicine, Department of Anatomy, Dicle University, Diyarbakir 21090, Turkey

**Keywords:** stroke, thrombectomy, nutritional status, prognosis, biomarkers

## Abstract

Background/objectives: Nutritional status is increasingly acknowledged as a pivotal determinant of clinical course and recovery in patients with acute ischemic stroke (AIS). Malnutrition can compromise immune competence, delay neurological recovery, and exacerbate adverse outcomes, particularly in those undergoing intensive interventions such as mechanical thrombectomy (MT). To objectively assess nutritional status, indices such as the Prognostic Nutritional Index (PNI) and the Controlling Nutritional Status (CONUT) score have been utilized in various clinical populations. These indices incorporate routinely available laboratory parameters, reflecting both nutritional and inflammatory states. This study explores whether PNI and CONUT scores are associated with 90-day clinical outcomes in AIS patients treated with MT, aiming to evaluate their potential utility as prognostic biomarkers in acute stroke care. Methods: A total of 404 patients with AIS who underwent MT between 2023 and 2024 were retrospectively evaluated. Demographic, clinical, and laboratory data were collected, and nutritional status was assessed using PNI and CONUT scores. Clinical outcomes were stratified as favorable (modified Rankin Scale [mRS] 0–2) or unfavorable (mRS 3–6) at 90 days post-stroke. Results: Among the 404 patients included in the study, 50.5% had favorable and 49.5% had unfavorable clinical outcomes. Patients with favorable outcomes were younger (71 vs. 78 years, *p* = 0.001), had lower National Institutes of Health Stroke Scale (NIHSS) scores, and higher Alberta Stroke Program Early CT Scores (ASPECTS) (*p* = 0.001). The puncture-to-recanalization time was significantly longer in the unfavorable outcome group (47.5 min vs. 30.0 min, *p* = 0.003). Laboratory findings revealed higher glucose levels (*p* = 0.029), and lower serum albumin (*p* = 0.003) and lymphocyte levels (*p* = 0.001) in the unfavorable outcome group. Among nutritional indices, the CONUT score was significantly higher in the unfavorable outcome group (*p* = 0.001), whereas the PNI score was higher in the favorable outcome group (*p* = 0.001). ROC analysis showed that the CONUT score had significant prognostic performance (AUC = 0.721, *p* < 0.001), while the PNI had poor discriminatory power (AUC = 0.274, *p* < 0.001). Multivariate logistic regression identified age, baseline NIHSS, ASPECT score, and CONUT score as independent predictors of clinical outcome (*p* < 0.05). Conclusions: Among the two nutritional indices evaluated, the CONUT score demonstrated significant prognostic value in predicting 90-day clinical outcomes after MT. In contrast, the PNI showed limited discriminatory power, highlighting the superiority of CONUT as a reliable biomarker in acute stroke care.

## 1. Introduction

Stroke is not only one of the leading causes of mortality worldwide but also the primary cause of disability, imposing significant social and economic burdens [1]. In recent years, outcomes in ischemic stroke have markedly improved due to the establishment of stroke units and the implementation of endovascular therapy for patients presenting with large vessel occlusions [2,3,4,5,6]. Numerous multiparametric models have been developed to predict favorable outcomes following endovascular stroke treatment; however, many of these models incorporate confounding factors such as time to treatment or the success of recanalization. Despite timely intervention and successful reperfusion, long-term clinical outcomes often fall short of expectations [7,8]. This discrepancy has led to the investigation of additional prognostic factors influencing post-stroke recovery.

In recent years, the role of nutritional status in determining clinical outcomes has garnered increasing attention. Nonetheless, there remains a paucity of data regarding whether the nutritional condition of patients undergoing MT impacts clinical outcomes. Few studies have evaluated nutritional indices specifically in MT patients, despite the widespread and increasing use of mechanical thrombectomy in acute stroke treatment protocols. This growing evidence has emphasized the importance of nutritional evaluation in AIS patients. However, most of the current literature focuses on general stroke populations or those treated with intravenous thrombolysis, while the nutritional status and its prognostic relevance in patients undergoing mechanical thrombectomy remain underexplored. Recent advances have highlighted the importance of nutritional status as a prognostic determinant in stroke recovery. Among various tools, the CONUT score has been increasingly utilized due to its simplicity and objectivity in capturing malnutrition-related immune suppression and metabolic dysfunction. Kokura et al. conducted a multicenter retrospective cohort study involving 702 adult stroke patients and found that a high CONUT score on admission, indicative of undernutrition, was independently associated with poorer improvement in motor functional independence as measured by FIM gain. Interestingly, this association was not observed for cognitive functional improvement. Their findings underscore the prognostic utility of nutritional status in stroke rehabilitation, particularly in motor recovery domains [9]. However, while several studies have evaluated the role of CONUT in general stroke populations, there remains a paucity of data specifically investigating its predictive role in AIS patients undergoing MT, a subgroup with unique clinical and procedural characteristics. Our study aims to address this knowledge gap.

According to the European Society for Clinical Nutrition and Metabolism (ESPEN), malnutrition is defined as a condition resulting from inadequate intake or absorption of nutrients, leading to changes in body composition—particularly a reduction in lean body mass and cellular mass—which ultimately impairs physical and mental function and worsens clinical outcomes in disease states [10]. Poor nutritional status adversely affects the diagnosis, prognosis, and clinical progression of numerous acute and chronic conditions [11]. The impact of malnutrition on disease progression is multifactorial: it causes reductions in organ mass and composition, compromising multiple physiological functions, and impairs immune responses and tissue repair processes [12]. Malnutrition is a common issue among stroke patients. Studies have reported a malnutrition prevalence of approximately 33–34.3% at hospital admission in patients with AIS [13,14]. Recent research has demonstrated that malnutrition at admission is associated with increased mortality and worse functional outcomes in AIS patients [15,16]. Therefore, early screening for nutritional status at hospital admission is crucial for enabling timely and effective nutritional interventions.

Assessing the nutritional status of patients remains challenging. Many nutritional indicators are subjective, as they rely on clinician experience or patient and caregiver reports. An initial realistic step in clinical practice would be to adopt validated, rapid, cost-effective, and reliable tools to assess nutritional risk. Several screening tools have been proposed and applied in stroke patients globally [17]. In particular, objective nutritional indices such as CONUT score and the PNI, which are calculated using serum albumin, cholesterol levels, and peripheral lymphocyte counts, offer a practical and accessible approach in routine clinical settings [18,19]. One study using these malnutrition indices found that higher malnutrition risk in ischemic stroke patients was associated with increased long-term mortality and severe disability [20]. However, the prognostic significance of nutritional status in patients undergoing intravenous thrombolysis (IVT) remains inadequately addressed.

The potential influence of nutritional status and immune response on post-stroke outcomes has increasingly attracted scientific interest. Given that the CONUT score and PNI reflect nutritional condition and immune competence, we hypothesized that these indices could serve as prognostic markers in patients with AIS undergoing MT. However, our review of the current literature revealed limited data and a lack of comprehensive studies evaluating the association between nutritional indices and clinical outcomes following MT. Accordingly, this study aims to investigate the relationship between the CONUT score and PNI with 90-day clinical outcomes in patients who underwent recanalization via MT, thereby providing valuable insights into their prognostic utility in acute stroke care. We hypothesized that both the CONUT score and PNI are associated with 90-day functional outcomes in patients with acute ischemic stroke undergoing mechanical thrombectomy.

## 2. Materials and Methods

### 2.1. Study Design

**Figure 1:**
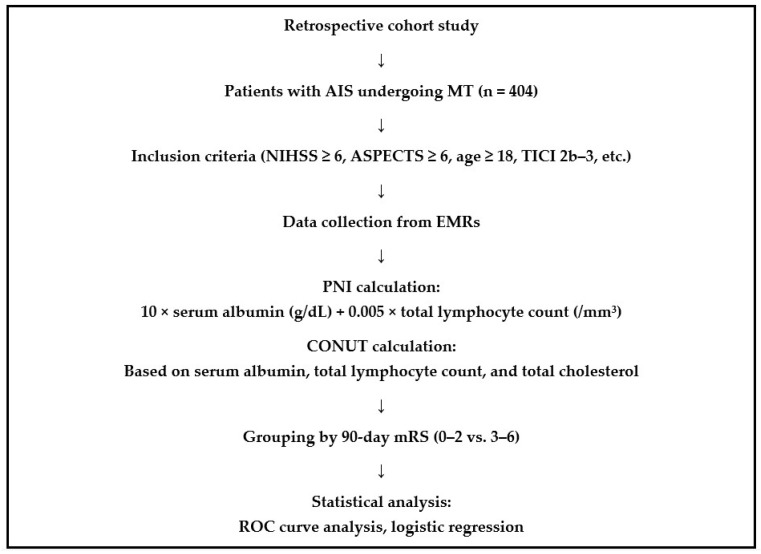
Flowchart illustrating the design and methodology of a retrospective cohort study involving 404 patients with acute ischemic stroke (AIS) who underwent mechanical thrombectomy (MT). The flowchart outlines key steps, including patient inclusion criteria (NIHSS ≥ 6, ASPECTS ≥ 6, age ≥ 18, TICI 2b–3), data collection from electronic medical records (EMRs), assessment of nutritional status using the Prognostic Nutritional Index (PNI) and Controlling Nutritional Status (CONUT) score, patient grouping based on 90-day modified Rankin Scale (mRS) outcomes (0–2 vs. 3–6), and statistical analyses, including ROC curve and logistic regression modeling.

This study was designed as a retrospective cohort analysis involving patients with AIS who underwent MT. Data were collected from patients treated between 2023 and 2024. Demographic, clinical, and laboratory data were retrospectively retrieved from electronic medical records (Figure 1). The study was approved by the Clinical Research Ethics Committee of the University of Health Sciences, Gazi Yaşargil Training and Research Hospital (Approval No: 389; 14 March 2025), and was conducted in accordance with the principles of the Declaration of Helsinki. Informed consent was waived due to the retrospective character of the analysis of anonymized patient data retrieved from electronic medical records. No direct patient contact or intervention was conducted, and all analyses were performed using de-identified information.

### 2.2. Inclusion Criteria

Patients were eligible for inclusion if they met all of the following criteria:

- Diagnosed with AIS due to large artery occlusion, confirmed by cranial CT angiography, cranial MRI angiography, or digital subtraction angiography;
- Presented to the hospital within 24 h of symptom onset;
- NIHSS score ≥ 6 on admission;
- ASPECTS ≥ 6;
- No evidence of intracranial hemorrhage on initial CT or MRI;
- Underwent mechanical thrombectomy within 6 h of symptom onset, or between 6 and 24 h in accordance with the eligibility criteria of the DAWN or DEFUSE trials;
- Achieved successful reperfusion, defined as a post-thrombectomy Thrombolysis in Cerebral Infarction (TICI) score of 2b-3;
- Pre-stroke mRS score between 0 and 2;
- Age 18 years or older.

### 2.3. Exclusion Criteria

- Patients with incomplete clinical or laboratory data

### 2.4. Data Collection

Demographic, clinical, and laboratory data of patients with AIS who underwent MT were retrospectively analyzed through electronic medical records. Collected baseline characteristics included age, sex, stroke onset time, NIHSS and ASPECTS scores at admission, comorbidities, vascular occlusion sites, and stroke subtypes. Procedural details such as time from symptom onset to groin puncture (onset-to-puncture time), time from puncture to recanalization (puncture-to-recanalization time), thrombectomy technique used, number of passes performed, administration of IV-tPA, and postprocedural TICI scores were recorded. Laboratory parameters included serum albumin, total lymphocyte count, total cholesterol, glucose, creatinine, hemoglobin, hematocrit, and LDL (mg/dL). Imaging and laboratory tests were performed in accordance with standard protocols upon admission. Patients with missing laboratory values necessary for calculating PNI or CONUT scores (such as serum albumin, lymphocyte count, or total cholesterol) were excluded from the nutritional index analysis. However, the exact number of excluded patients due to missing data was not separately recorded during retrospective data collection.

### 2.5. Calculation of Nutritional Indices

Nutritional status was evaluated using the PNI and CONUT scores.

#### 2.5.1. PNI Calculation

PNI was calculated using the following formula based on serum albumin and total lymphocyte count:

PNI = Serum Albumin (g/dL) + (5 × Total Lymphocyte Count [×10⁹/L])

PNI scores were used to assess the nutritional risk of patients.

#### 2.5.2. CONUT Calculation

The CONUT score was derived from serum albumin, total lymphocyte count, and total cholesterol levels. Each parameter was scored individually, and the total score was calculated as follows:

Serum Albumin (g/dL): ≥3.5 (0 points), 3.0–3.4 (2 points), 2.5–2.9 (4 points), <2.5 (6 points).

Total Lymphocyte Count (×10⁹/L): ≥1600 (0 points), 1200–1599 (1 point), 800–1199 (2 points), <800 (3 points).

Total Cholesterol (mg/dL): ≥180 (0 points), 140–179 (1 point), 100–139 (2 points), <100 (3 points).

It should be noted that all laboratory parameters used for nutritional index calculation (albumin, total lymphocyte count, and total cholesterol) were obtained at the time of hospital admission, prior to mechanical thrombectomy. Although these values reflect baseline nutritional and inflammatory status, they may be influenced by acute-phase responses and hemodynamic fluctuations following MT, which could affect their stability over time.

### 2.6. Clinical Outcome Assessment

Clinical outcomes were evaluated on day 90 using mRS, a standardized tool ranging from 0 (no symptoms) to 6 (death) used to assess the degree of functional recovery. An mRS score of 0–2 was defined as a “favorable clinical outcome,” whereas a score of 3–6 was classified as an “unfavorable clinical outcome.” Outcome data were obtained from medical records and/or direct patient interviews.

### 2.7. Statistical Analysis

All statistical analyses were performed using IBM SPSS Statistics version 25.0. The distribution of continuous variables was assessed using the Shapiro–Wilk test. Normally distributed data were expressed as mean ± standard deviation (SD), whereas non-normally distributed data were reported as median with interquartile range (IQR). Categorical variables were expressed as frequencies and percentages. For comparisons between groups, the chi-square or Fisher’s exact test was used for categorical variables (depending on expected cell counts), the independent samples t-test for normally distributed continuous variables, and the Mann–Whitney U test for non-normally distributed variables.

Multivariate logistic regression analysis was performed to identify independent prognostic factors associated with favorable outcomes. Associations were reported as odds ratios (ORs) with 95% confidence intervals (CIs). To assess the predictive accuracy of the nutritional indices, the Receiver Operating Characteristic (ROC) curve analysis was conducted. The area under the curve (AUC) was calculated to compare the predictive performance of the CONUT and PNI scores in determining clinical outcomes. A *p*-value < 0.05 was considered statistically significant for all analyses.

Variables included in the multivariate model were selected based on univariate significance and clinical relevance. However, due to the retrospective nature of the study and collinearity concerns, potential confounding factors such as the presence of diabetes mellitus were not adjusted separately in glucose-outcome association models.

## 3. Results

### 3.1. Clinical Outcomes and Baseline Characteristics

In this study, 404 patients with AIS who underwent MT were analyzed. Among these, 204 patients (50.5%) achieved favorable clinical outcomes, while 200 patients (49.5%) had poor outcomes. The median age was significantly lower in the favorable outcome group [71 (59.00–78.00)] compared to the poor outcome group [78 (71.25–84.00)] (*p* = 0.001). Regarding sex distribution, 44.6% of the favorable outcome group were female compared to 54.0% in the poor outcome group, though this difference was not statistically significant (*p* = 0.194). No significant differences were found between the groups in terms of comorbidities such as hypertension, diabetes mellitus, atrial fibrillation, hyperlipidemia, coronary artery disease, or heart failure (*p* > 0.05) (Table 1).

### 3.2. Stroke Severity, Imaging Scores, and Procedural Times

The NIHSS score was significantly lower in the favorable outcome group [median 13 (10.00–17.50)] than in the poor outcome group [median 17 (14.00–22.00)] (*p* = 0.001). Similarly, the ASPECTS was significantly higher in the favorable outcome group (9 vs. 7, *p* = 0.001). No significant difference was found in symptom-to-puncture time (*p* = 0.106); however, the puncture-to-recanalization time was significantly shorter in the favorable group [30.00 min vs. 47.50 min, *p* = 0.003].

### 3.3. Stroke Etiology and Vascular Occlusion Sites

There were no significant differences between groups in stroke etiology: cardioembolism (55.9% vs. 56.5%), large artery atherosclerosis (19.6% vs. 28.0%), or other causes (*p* > 0.05). The most common site of occlusion was MCA M1 (47.5% vs. 50.0%), with no significant difference between groups (*p* = 0.619).

### 3.4. Recanalization and Procedure Parameters

A significantly higher proportion of patients in the favorable outcome group achieved complete recanalization (TICI 3) compared to the poor outcome group (67.2% vs. 31.5%, *p* = 0.001). Thrombectomy technique distribution (Solumbra, ADAPT, and Solumbra+ADAPT) did not differ significantly (*p* = 0.736). The number of thrombectomy passes was lower in the favorable outcome group [median 1 (1–2) vs. 2 (1–3), *p* = 0.001].

### 3.5. Laboratory Findings and Nutritional Indices

Baseline laboratory parameters and nutritional scores are summarized in Table 2. Blood glucose levels were significantly higher in the poor outcome group [154.0 mg/dL (120.0–196.0)] compared to the favorable group [127.0 mg/dL (114.0–155.5)] (*p* = 0.029). Serum albumin levels were higher in the favorable outcome group [38 g/dL (36–40) vs. 36 g/dL (30–39), *p* = 0.003]. Lymphocyte counts were also significantly elevated in the favorable group [1.99 × 10^3^/µL (1.28–3.30)] vs. [1.29 × 10^3^/µL (0.86–2.05), *p* = 0.001]. The PNI was significantly higher in the favorable outcome group (49.7 ± 7.75 vs. 42.8 ± 8.97, *p* = 0.001), whereas the CONUT score was significantly higher in the poor outcome group [2.5 (2–5) vs. 1 (1–3), *p* = 0.001].

### 3.6. Predictive Value of Nutritional Indices: ROC Analysis

**Figure 2:**
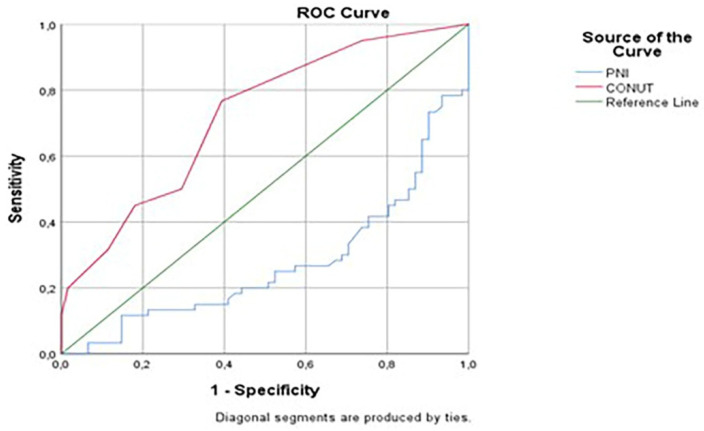
Receiver Operating Characteristic (ROC) curves for CONUT and PNI scores in predicting unfavorable clinical outcomes. The ROC curve demonstrates the diagnostic performance of the Controlling Nutritional Status (CONUT) score and the Prognostic Nutritional Index (PNI) in predicting unfavorable clinical outcomes (mRS 3–6) at 90 days following mechanical thrombectomy. The area under the curve (AUC) was 0.721 for CONUT (*p* < 0.001) and 0.274 for PNI (*p* < 0.001). The diagonal green line represents the reference line (AUC = 0.5), indicating no discriminatory power. The CONUT score shows superior predictive performance compared to the PNI.

ROC analysis revealed that the CONUT score had an AUC of 0.721 (95% CI: 0.631–0.811; *p* < 0.001), demonstrating acceptable discriminative ability for predicting functional independence at 90 days. In contrast, the PNI yielded an AUC of 0.274 (95% CI: 0.184–0.364; *p* = 0.001), suggesting not only poor but inverse predictive value, meaning that higher PNI scores were paradoxically associated with worse outcomes. This inversion highlights the need for careful interpretation and potential reconsideration of PNI’s standalone use in acute stroke settings involving mechanical thrombectomy (Figure 2). Given its extremely poor performance and inverse predictive direction, the PNI was not included in the multivariate regression model. Including a variable with such characteristics could potentially distort the regression model and reduce the interpretability of other covariates. Therefore, only the CONUT score, which demonstrated meaningful prognostic value, was retained for further modeling.

At an optimal cut-off point of 3.5 for the CONUT score, the sensitivity and specificity for predicting 90-day functional independence were approximately 85% and 68%, respectively. These values indicate that the CONUT score offers a reasonable balance between identifying patients at risk for poor outcomes and minimizing false-positive predictions, reinforcing its potential clinical utility as a prognostic marker in AIS patients undergoing mechanical thrombectomy.

### 3.7. Independent Predictors of Clinical Outcome: Multivariate Analysis

Multivariate logistic regression analysis results are presented in Table 3. Admission NIHSS score was significantly associated with poor clinical outcome (*p* = 0.005), where each one-point increase decreased the likelihood of a favorable outcome by 11.9% (OR: 0.881, 95% CI: 0.807–0.962). Higher ASPECTS scores were strongly associated with favorable outcomes (*p* < 0.001), with each unit increase raising the odds by approximately 3.4 times (OR: 3.414, 95% CI: 2.022–5.764). Age was negatively associated with favorable outcomes (*p* = 0.020), with each additional year reducing the odds by 5.5% (OR: 0.945, 95% CI: 0.901–0.991). Similarly, higher CONUT scores were associated with worse outcomes (*p* = 0.034), where each unit increase reduced the likelihood of a favorable outcome by 27.2% (OR: 0.728, 95% CI: 0.543–0.976). Other variables, including puncture-to-recanalization time, number of passes, and glucose levels, did not show significant independent associations with clinical outcome (*p* > 0.05).

## 4. Discussion

This study was among the first to evaluate the association between nutritional status and clinical outcomes in patients with AIS undergoing MT. The findings demonstrated that the CONUT score serves as an independent prognostic indicator for poor clinical outcomes, whereas PNI exhibited limited predictive power. In addition to traditional prognostic markers such as age, number of thrombectomy passes, NIHSS, and ASPECTS scores, nutritional status was identified as a significant determinant of post-MT recovery.

The significant association between a higher CONUT score and poor clinical outcomes (OR: 0.728, *p* = 0.034) highlights the integrated role of nutritional and immune status in stroke prognosis. The CONUT score, by incorporating serum albumin, total cholesterol, and lymphocyte count, serves as a multidimensional index that reflects both malnutrition and systemic inflammation [21,22]. This aligns with previous studies showing that elevated CONUT scores are linked to increased mortality and disability in general stroke populations [23,24]. For instance, high CONUT scores have been independently associated with worse 3-month mRS scores in ischemic stroke patients [25]. However, this study was among the first to validate the prognostic utility of the CONUT score in patients who achieved recanalization following MT. The superior predictive performance of CONUT compared to PNI in this population (AUC: 0.721 vs. 0.274) may be partially attributable to the inclusion of cholesterol, which is known to fluctuate during acute-phase responses and may be indirectly influenced by inflammation or metabolic alterations [26]. Additionally, low serum albumin levels are known to exacerbate endothelial dysfunction and neuroinflammation [27], potentially explaining the stronger association between the CONUT score and clinical outcomes following MT. 

Although the PNI is a commonly used index reflecting nutritional and immune status, it demonstrated poor and inverse predictive performance in our cohort, with an AUC of 0.274. This value indicates that higher PNI scores were paradoxically associated with poorer clinical outcomes, suggesting a potential reversal in its expected prognostic role. Such findings may stem from the dynamic metabolic and inflammatory fluctuations occurring during acute ischemia-reperfusion injury. Albumin synthesis is suppressed during the acute phase, and vascular permeability increases, leading to reductions in serum levels. Lymphopenia, on the other hand, reflects immunosuppression due to sympathetic activation and cortisol elevation [28,29]. Despite blood sampling prior to reperfusion, these transient changes under acute stress may have undermined the stability of PNI and rendered it a misleading prognostic tool in this population. Therefore, caution is warranted when interpreting PNI in hyperacute stroke settings. Future studies may explore whether serial measurements or integration with complementary biomarkers could improve its prognostic accuracy [19]. In dynamic conditions such as AIS, where inflammation occurs rapidly and intensely, the clinical utility of PNI may be limited. Future studies may explore whether serial PNI measurements or integration with other biomarkers could improve its prognostic value, although its standalone utility appears limited in acute stroke settings. Additionally, future research should focus on developing dynamic indices that more accurately reflect the evolving inflammatory and nutritional status in order to enhance clinical outcome prediction in patients with acute ischemic stroke.

Interestingly, the puncture-to-recanalization time did not emerge as a significant factor in multivariate regression analysis (*p* > 0.05), partially contradicting previous studies [30]. However, the narrow time range observed in this cohort (median 30–47.5 min) and uniform achievement of TICI 2b-3 recanalization may have masked this variable’s effect. Similarly, although elevated glucose levels were associated with poor outcomes—possibly due to the exacerbation of ischemia-reperfusion injury by hyperglycemia—they did not retain statistical significance in the multivariate analysis, potentially due to unadjusted confounding from metabolic conditions such as diabetes mellitus, which strongly influences baseline glucose levels [31].

One of the main limitations of this study is its retrospective design. Data were collected from a single center, which increases the risk of selection bias and limits the generalizability of the findings. Furthermore, nutritional parameters such as serum albumin, lymphocytes, and total cholesterol were assessed at a single time point, which may not adequately reflect the dynamic nutritional status during the acute phase. These biomarkers can be temporarily altered by acute-phase reactions such as inflammation and stress-induced hyperglycemia, possibly misrepresenting the true nutritional status. Another limitation is that the study population included only patients who achieved successful recanalization (TICI 2b-3). This homogenous cohort reflects high procedural success, but the prognostic value of nutritional indices remains unknown in patients with unsuccessful recanalization or lower TICI scores. Moreover, the exclusion of patients with incomplete laboratory data may have introduced selection bias, as those with missing values might differ systematically from those included in the final analysis. Lastly, missing laboratory data (e.g., unrecorded cholesterol or lymphocyte levels in some patients) due to the retrospective nature of data collection may have reduced statistical power and introduced uncertainty in the interpretation of results. To address these limitations and to confirm the generalizability and prognostic value of nutritional indices such as CONUT and PNI, we recommend a prospective, multicenter validation cohort in future studies.

The results of this study underscore the importance of incorporating nutritional assessment into the routine evaluation of AIS patients undergoing MT. The CONUT score, as a simple, cost-effective, and routinely available tool, may provide valuable prognostic information beyond conventional clinical and radiological parameters. Early identification of patients with poor nutritional and immunological profiles could facilitate timely interventions aimed at optimizing metabolic and inflammatory status during the acute phase of stroke recovery. Such interventions may include tailored nutritional support, anti-inflammatory strategies, or intensified monitoring in high-risk individuals. By integrating nutritional indices into comprehensive stroke care pathways, clinicians may enhance individualized patient management and potentially improve post-thrombectomy functional outcomes.

Given the prognostic significance of the CONUT score observed in this study, future prospective, multicenter trials are warranted to validate its predictive value in diverse stroke populations, including those with unsuccessful recanalization or lower TICI scores. Moreover, serial assessment of nutritional markers over time may provide dynamic insights into the evolution of nutritional status and its interaction with inflammatory and metabolic responses during the stroke continuum. Combining CONUT with additional biomarkers such as C-reactive protein, neutrophil-to-lymphocyte ratio, or interleukin-6 may further enhance prognostic precision. Development of refined, composite indices that integrate nutritional, inflammatory, and metabolic domains could better capture the pathophysiological complexity of acute stroke and guide personalized therapeutic strategies. Ultimately, interventional studies investigating whether targeted nutritional optimization can translate into improved clinical outcomes are needed to establish the clinical utility of such indices in routine practice.

## 5. Conclusions

This study is among the first to evaluate the association between nutritional status and clinical outcomes in patients with AIS undergoing MT, demonstrating that the CONUT score (AUC: 0.721) is a valuable independent prognostic indicator. In addition to traditional prognostic factors such as age, number of thrombectomy passes, NIHSS score, and ASPECTS, the study emphasizes the predictive utility of early nutritional assessment using the CONUT score. However, several limitations must be acknowledged, including the retrospective design, single-center data collection, and assessment of nutritional parameters at a single time point, which may restrict the generalizability of the findings. Prospective multicenter studies with serial nutritional assessments are needed to clarify the temporal relationship between malnutrition, inflammation, and post-thrombectomy recovery. Furthermore, the development of artificial intelligence–assisted multiparametric models may contribute to the optimization of personalized therapeutic strategies in acute stroke management.

## Figures and Tables

**Table 1:** Comparison of demographic, medical, and clinical characteristics between patients with favorable and unfavorable clinical outcomes. Values are presented as median (interquartile range) or number (%). Comparisons were made using the Mann–Whitney U test for continuous variables and the chi-square test or Fisher’s exact test for categorical variables, where appropriate. A *p*-value < 0.05 was considered statistically significant and is marked with an asterisk (*).

Variable	Favorable Outcome (N = 204)	Unfavorable Outcome (N = 200)	*p*-Value
Age, Median (IQR)	71 (59.00–78.00)	78 (71.25–84.00)	0.001 *
Sex, n (%)			
Female	91 (44.6)	108 (54.0)	0.194
Male	113 (55.4)	92 (46.0)	
Medical history, n (%)			
Hypertension	157 (77.0)	163 (81.5)	0.654
Diabetes Mellitus	63 (30.9)	80 (40.0)	0.346
Atrial Fibrillation	100 (49.0)	110 (55.0)	0.522
Hyperlipidemia	80 (39.2)	70 (35.0)	0.304
Coronary Artery Disease	97 (47.5)	107 (53.5)	0.587
Heart Failure	43 (21.1)	47 (23.5)	0.830
Previous Stroke	33 (16.2)	50 (25.0)	0.241
Malignancy	7 (3.3)	7 (3.5)	0.987
Smoking	63 (30.9)	53 (26.5)	0.689
Alcohol	20 (9.8)	17 (8.5)	0.744
Clinical features			
Intravenous Thrombolysis	100 (49.01)	85 (42.5)	0.255
NIHSS, Median (IQR)	13 (10.00–17.50)	17 (14.00–22.00)	0.001 *
ASPECTS, Median (IQR)	9 (9–10)	7 (7–8)	0.001 *
Symptom-to-Puncture Time (min), Median (IQR)	240 (150.00–312.50)	275 (200.00–360.00)	0.106
Puncture-to-Recanalization Time (min), Median (IQR)	30.00 (25.00–50.00)	47.50 (30.00–87.50)	0.003 *
Stroke Type, n (%)			
Cardioembolism	114 (55.9)	113 (56.5)	0.305
Large Artery Atherosclerosis	40 (19.6)	56 (28.0)	
Other	50 (24.5)	30 (15.0)	
Vascular Occlusion Site, n (%)			
MCA M1	97 (47.5)	100 (50.0)	0.619
MCA M2	30 (14.7)	17 (8.5)	
ICA T/L	20 (9.8)	47 (23.5)	
Basilar	27 (13.2)	17 (8.5)	
PCA	7 (3.3)	0 (0)	
Tandem	23 (11.3)	20 (10.0)	
TICI Score, n (%)			
2b	20 (9.8)	63 (31.5)	0.001 *
2c	47 (23.0)	73 (36.5)	
3	137 (67.2)	63 (31.5)	
Thrombectomy Technique, n (%)			
Solumbra	147 (72.1)	150 (75.0)	0.736
ADAPT	30 (14.7)	30 (15.0)	
Solumbra + ADAPT	27 (13.2)	20 (10.0)	
Number of Passes, Median (IQR)	1 (1–2)	2 (1–3)	0.001 *

**Table 2:** Laboratory parameters and nutritional scores at admission according to clinical outcome. Values are expressed as median (interquartile range) or mean ± standard deviation, depending on distribution. The Mann–Whitney U test was used for non-normally distributed variables, and the independent *t*-test was used for normally distributed variables. A *p*-value < 0.05 was considered statistically significant and is denoted by an asterisk (*).

Parameter	Favorable Outcome (N = 204)	Unfavorable Outcome (N = 200)	*p*-Value
Glucose (mg/dL), Median (IQR)	127.0 (114.0–155.5)	154.0 (120.0–196.0)	0.029 *
Creatinine (mg/dL), Median (IQR)	0.82 (0.69–1.00)	0.85 (0.68–1.27)	0.469
Hemoglobin (g/dL), Mean ± SD	13.48 ± 2.33	12.70 ± 2.10	0.057
LDL (mg/dL), Mean ± SD	108.14 ± 30.94	98.61 ± 30.76	0.092
Total Cholesterol (mg/dL), Mean ± SD	171.77 ± 35.56	160.63 ± 36.71	0.093
Serum Albumin (g/dL), Median (IQR)	38 (36–40)	36 (30–39)	0.003 *
Lymphocytes (10^3^/µL), Median (IQR)	1.99 (1.28–3.30)	1.29 (0.86–2.05)	0.001 *
Prognostic Nutritional Index (PNI), Mean ± SD	49.7 ± 7.75	42.8 ± 8.97	0.001 *
Controlling Nutritional Status (CONUT), Median (IQR)	1 (1–3)	2.5 (2–5)	0.001 *

**Table 3:** Multivariate logistic regression analysis of factors associated with clinical outcome. Odds ratios (OR) and 95% confidence intervals (CI) are reported. A *p*-value < 0.05 was considered statistically significant and is indicated by an asterisk (*). Variables were included in the model based on univariate significance or clinical relevance.

Variables	OR (95% CI)	*p*-Value
Admission NIHSS	0.881 (0.807–0.962)	0.005 *
ASPECT Score	3.414 (2.022–5.764)	0.001 *
Puncture-to-Recanalization Time	1.007 (0.984–1.031)	0.556
Number of Passes	0.460 (0.194–1.095)	0.039 *
Glucose	0.995 (0.985–1.006)	0.387
Age	0.945 (0.901–0.991)	0.020 *
CONUT Score	0.728 (0.543–0.976)	0.034 *

## Data Availability

All details about the study can be obtained from the corresponding author.
